# Characterization of an At-Risk Population for Nonalcoholic Fatty Liver Disease (NAFLD) in a Primary Care Setting Along the U.S.–Mexico Border

**DOI:** 10.1177/10436596241271265

**Published:** 2024-08-27

**Authors:** Lindsay Spitz, Stefan Saadiq, Navkiran K. Shokar, Marc J. Zuckerman, Nancy A. Casner, Roy Valenzuela, Jennifer J. Salinas

**Affiliations:** 1 Texas Tech University Health Sciences Center, El Paso, USA; 2 The University of Texas at Austin, USA; 3 The University of Texas at EL Paso, USA

**Keywords:** liver diseases, fatty liver, primary health care, evidence-based medicine, diagnostic tests, routine

## Abstract

**Introduction:**

This study aimed to determine the burden of suspected nonalcoholic fatty liver disease (NAFLD) and nonalcoholic steatohepatitis (NASH) in a predominantly Hispanic patient population and explore the utility of the American Gastroenterological Association’s NAFLD Clinical Care Pathway (CCP).

**Methodology:**

Electronic medical records (*n* = 223) were used to divide patients into risk groups based on the amount of metabolic risk factors they presented, diabetic status, or if they presented other liver diseases. Fribosis-4 (FIB-4) scores were used to determine the risk for advanced fibrosis.

**Results:**

Most patients (83.8%) were considered at risk for NAFLD based on CCP criteria, and about a third of patients (33.2%) were found to be at indeterminate (*n* = 60; 26.9%) or high risk (*n* = 14; 6.3%) for advanced fibrosis. Most indeterminate-risk patients (78.3%) were not referred for liver imaging.

**Discussion:**

This study demonstrates the potential of the CCP as a corrective tool that could help to better identify and screen patients at risk for NAFLD.

## Introduction

Nonalcoholic fatty liver disease (NAFLD), a spectrum of liver conditions characterized by the presence of hepatic steatosis without a secondary explanation (heavy alcohol use, other liver conditions, medication), is estimated to affect approximately 25% of the global population (Younossi et al., 2016). NAFLD is strongly associated with visceral obesity, type 2 diabetes, and metabolic risk factors and is rising at rates proportional to obesity and metabolic syndrome in Western countries (Al-Dayyat et al., 2018; Juanola et al., 2021; Kanwal et al., 2020; Younossi et al., 2016, 2018). Consequences of the disease include an increased risk of hepatocellular carcinoma (HCC) and elevated rates of liver-specific, cardiovascular-specific, and all-cause mortality (Ong et al., 2008; Younossi et al., 2019). These risks are further elevated in cases where NAFLD has advanced from benign steatosis to nonalcoholic steatohepatitis (NASH), a condition characterized by liver inflammation and hepatocyte injury. NASH is estimated to affect between 3% and 6% of the U.S. population and is more likely to progress to advanced fibrosis, cirrhosis, and end-stage liver disease than NAFLD alone (Al-Dayyat et al., 2018; Estes et al., 2018; Juanola et al., 2021; Kanwal et al., 2020; Ong et al., 2008; Younossi et al., 2016, 2018, 2019).

NAFLD prevalence in the United States is highest among the Hispanic population when compared to other races/ethnicities, with those of Mexican descent displaying the highest rates of NAFLD of any Hispanic subpopulation and should be of particular concern to physicians and nurses practicing in U.S.–Mexico border settings (Fleischman et al., 2014; Garcia et al., 2022; Rich et al., 2018; Samji et al., 2020). The U.S. Hispanic population experiences high rates of obesity, metabolic risk factors, and type 2 diabetes, all of which are associated with an increased risk of NAFLD. Genetic predisposition may also be partially responsible, as an allelic variation in the patatin-like phospholipase domain-containing protein 3 (PNPLA3) associated with increased liver fat occurs more frequently in the U.S. Hispanic population (Fleischman et al., 2014; Garcia et al., 2022; Samji et al., 2020). The Mexican subpopulation displays the highest proportion of PNPLA3 variation and alternative genotypes in additional genetic markers (e.g., LYPLAL1, PPP1R3B, GCKR) associated with an increased risk of NAFLD (Larrieta-Carrasco et al., 2018; Rich et al., 2018; Samji et al., 2020). Furthermore, Hispanic patients with NAFLD experience an increased risk for progression to NASH, face the most significant burden of NAFLD-related HCC, and demonstrate the highest demand for liver transplants compared with other ethnicities (Couto et al., 2013; Ha et al., 2017; Samji et al., 2020).

Early identification of NAFLD reduces the likelihood of progression to NASH, advanced fibrosis, cirrhosis, and HCC (Kanwal et al., 2021; Zoubek et al., 2017). Liver fibrosis is a strong predictor of liver-specific disease outcomes and is used as a proxy to determine the severity of NAFLD progression. The most accurate method of fibrosis detection is liver biopsy; however, it carries several disadvantages. For instance, liver biopsy sampling can be inaccurate, costly, and can come with a potential risk of complications for the patient (Sumida et al., 2014). It is also not realistic to perform a liver biopsy on all NAFLD patients, which has led to the development of noninvasive alternatives (Loomba & Adams, 2020). These noninvasive tests use serum-based approaches, which measure blood-based biomarkers, or liver elastography techniques, which approximate liver stiffness, to assess liver fibrosis and stage NAFLD progression (Angulo et al., 2015; Castera et al., 2019; Unalp-Arida & Ruhl, 2017). Noninvasive biomarkers such as alanine aminotransferase (ALT) have been used to detect NAFLD; however, studies found that normal levels of ALTs are found in NAFLD patients as well (Sumida et al., 2014). In cases of advanced fibrosis, the FIB-4 index and the NAFLD fibrosis score (NFS), which are calculated using a biomarker scoring system, are helpful in ruling out NASH (Sumida et al., 2014).

Serum tests are adequate methods to rule out the presence of fibrosis and cirrhosis and can be further divided into two loose categories: simple and complex (Castera, 2020; Harman et al., 2015). Simple tests utilize inexpensive, widely available biomarkers acquired via standard panels that indirectly assess fibrosis. These include the aspartate aminotransferase/alanine aminotransferase (AST/ALT) ratio, FIB-4, NFS, and AST Platelet Ratio Index (APRI) (Castera, 2020; Castera et al., 2019; Lee et al., 2021; Vallet-Pichard, 2007; Wai et al., 2003; Xiao et al., 2017). In contrast, complex tests such as the Enhanced Liver Fibrosis (ELF) Score, Fibrospect II, and Fibrometer detect direct indicators of fibrogenesis and fibrinolysis using patented technologies (Calès et al., 2009; Huang et al., 2017; Loomba & Adamsc, 2020; Xiao et al., 2014; Xiao et al., 2017). As a result, the proprietary methods are the most accurate, while simple serum tests are the most accessible and cost-effective.

In cases where serum testing cannot rule out fibrosis, confirmation requires elastography, a screening technique that assesses liver stiffness. Transient elastography (Fibroscan), an ultrasound-based method, is the best-validated and most accessible elastography technology (Bloom et al., 2018; Loomba & Adams, 2020; Siddiqui et al., 2019). Magnetic resonance elastography, which combines magnetic resonance imaging (MRI) with low-frequency vibrations to create a visual map of tissue stiffness, provides more accurate results due to reductions in sampling error and technical failure rates (Loomba & Adams, 2020; Mayo Clinic, 2022; Singh et al., 2014; Wagner et al., 2017). Unfortunately, the technology is only accessible in very select clinics and is expensive, making population-level implementation unrealistic. In the case of population-based screening, there appears to be general agreement that a combination assessment, beginning with simple serum tests followed by more specialized testing and referral to elastography, is most appropriate (Bertrais et al., 2017; Boursier et al., 2017; Y. Patel et al., 2018; Petta et al., 2015; Petta et al., 2017).

Despite abundant noninvasive tests, NAFLD screening remains suboptimal (Blais et al., 2015; Castera et al., 2019; P. Patel et al., 2018). Without clear decision-making guidelines to make sense of available noninvasive tests, it is up to the individual primary care physician (PCP) or nurse practitioner to determine their NAFLD screening and referral process. Unfortunately, this leads to inconsistent approaches among PCPs and nurses, some of whom may entirely forgo adequate screening and subsequent referrals to follow-up assessment and specialist care (Blais et al., 2015). To remedy this, the American Gastroenterological Association (AGA) introduced a NAFLD Clinical Care Pathway (CCP), consolidating disparate clinical criteria and tests into a practical guide for NAFLD screening, diagnosis, and management (Kanwal et al., 2021). The pathway uses evidence-based decision points to stratify patients among low, indeterminate, and high-risk groups for which it provides care recommendations (Kanwal et al., 2021).

Proper NAFLD screening is of particular concern for U.S.–Mexico border communities, as their predominately Mexican-origin populace is at an increased risk for NAFLD and its complications. This study, which retrospectively applied the 2021 AGA CCP to primary care patient records predating pathway development, served as a hypothetical exercise to determine the potential impact of the pathway on NAFLD risk detection and the primary care screening practices in a U.S.–Mexico border setting. The research aims were to (a) identify the percentage of patients with at least one metabolic risk factor who met the criteria for screening and (b) determine to what extent PCPs and nurses adequately screened patients for NAFLD, based on the CCP guidelines, prior to pathway introduction. This exploratory application will allow researchers and clinicians to conceptualize the potential improvements to NAFLD risk detection and screening offered by the pathway. It will also inform future primary care providers of education regarding NAFLD screening and prospective study design.

## Method

### Setting

There are roughly 2.6 million Mexican Americans living in the 32 counties bordering Texas and Mexico (Valenzuela et al., 2022). El Paso, the location of this study, is the largest city in Texas along the U.S/Mexico border. El Paso has a population of 869,880 people, and the median household income is $55,417 and 21% of the population is considered poor (U.S. Census Bureau, 2023). Almost one-quarter (23%) of the population in El Paso was born outside the United States (DATAUSA, 2023). However, language is not viewed as a barrier in this area because most El Pasoans can speak English. A report by Adams (2023) indicated that almost 31% of El Pasoans spoke English only at home. A study for Stacker (2022) indicated that while most of the population in El Paso can speak Spanish (64%); about 57% (358,951) can also speak English very well and roughly 265,965 of them can speak it less than very well. Most El Pasoans (80%) have some type of health insurance and on average, physicians see about 2,003 patients per year (DataUSA). Roughly 40% of the residents in El Paso, Texas are also classified as being obese (Valenzuela et al., 2022). The border region of El Paso County encompasses roughly 1015^2^ miles and includes a population of 82.9% Hispanic/Latino, most of them being of Mexican American descent.

### Site Selection

El Paso was viewed as a great place to test the efficacy of the CCP due to the Hispanic population, which consisted of predominantly Mexican Americans. As previously noted, Mexican Americans are the race/ethnicity with the highest prevalence of NAFLD in the United States. They are also the population with the highest proportion of the PNPLA3 variation and alternative genotypes, which is a high-risk indicator of NAFLD. A conference call with Dr. Kanwal, one of the creators of the CCP, assured the authors that El Paso would serve as an adequate place to test the efficacy of the CCP. Another reason for the site selection was that the authors were permitted to access electronic medical records from Texas Tech University Health Sciences Center-El Paso.

### Data Extraction and Preparation

This university has a robust lineup of cancer prevention programs to address cancers common among Hispanic patients (Urbina, 2022). To date, combined programming has enrolled well over 32,000 patients, predominantly under and uninsured, most of whom are low-income, resulting in improved detection of breast and colorectal cancers, administration of cost-free HPV vaccines, and provision of wellness education for over 2,800 community members (Urbina, 2022; Valenzuela et al., 2021; Willauer et al., 2022). This study utilized patient records collected between 2016 and 2018 from adults with at least one visit to a TTUHSCEP-affiliated family medicine clinic in El Paso, TX, with a staff of 12 board-certified family medicine physicians. Patients with at least one NAFLD risk factor (obesity, diabetes, lipid disorder, metabolic disorder, or abnormal liver function tests) were identified from the EMR using ICD9 and ICD10 billing codes corresponding to metabolic risk factors (diabetes mellitus, obesity/overweight, hyperlipidemia, and metabolic syndrome) and liver abnormalities (fatty liver, NASH, cirrhosis, and abnormal AST or ALT).

Of the 10,000 cases identified, 500 were randomly selected for inclusion in the study, and 324 charts were ultimately abstracted due to a shortage of available abstractors resulting from the COVID-19 pandemic. Laboratory test results were extracted from the EMR to calculate the metrics assessed in the CCP. Of the 324 randomly selected EMRs, 306 met the requirement of containing a participant’s age, 244 EMRs met the requirement of including the complete blood count laboratory results, and 275 included a liver function test. This led to the exclusion of 101 records from the selected sample. Ultimately, the final sample contained 223 records with all necessary data for further analysis. Error in unit measurements and data entry were corrected by methods stated below. The TTUHSC El Paso Institutional Review Board approved this study.

### Description of the NAFLD CCP

**Figure 1.**
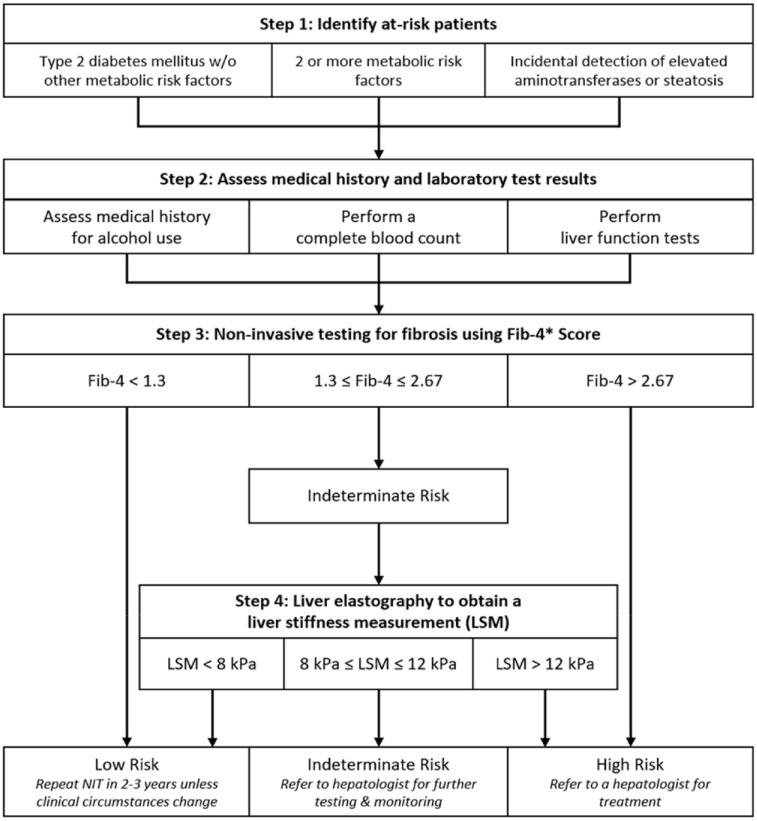
American Gastroenterological Association NAFLD CCP Screening Process to Detect Advanced Fibrosis Adapted From Kanwal et al. (2020).

The AGA’s NAFLD CCP comprises standardized recommendations for NAFLD screening based on disease risk (see Figure 1). The first step in the pathway identifies patients at risk for NAFLD with advanced fibrosis based on established risk factors (Kanwal et al., 2021). These include two or more metabolic risk factors, type 2 diabetes without additional metabolic risk factors, and incidental detection of elevated aminotransferase levels or observable liver steatosis.

The second step of the pathway aims to rule out alternative sources of liver disease via medical history and laboratory testing, namely a complete blood count (CBC) and liver function tests (Kanwal et al., 2021). Patients with a history of heavy alcohol use (≥14 drinks/week for women; ≥21 drinks/week for men), indicative of alcohol-related liver disease, or histology indicating alternative diagnoses should be referred to a hepatologist (Curry et al., 2018; Kanwal et al., 2021). Those for whom NAFLD has not been ruled out continue to Step 3, where test results from the previous step (AST, ALT, platelet count) and the patient’s age are used to calculate a Fibrosis-4 (FIB-4) score (Kanwal et al., 2021). This score indirectly approximates fibrosis severity and allows patient classification as low, indeterminate, or high risk based on established cutoffs (low: <1.3; indeterminate: 1.3–2.67; high: >2.67).

Those at low risk are advised to repeat the screening every 2–3 years until circumstances change, while those at high risk should be referred to a hepatologist immediately (Kanwal et al., 2021). Patients demonstrating indeterminate risk continue to the final step of the pathway, liver elastography, a screening technique that assesses liver stiffness. Those with liver stiffness measurements less than 8 kPa are considered low risk and may follow the low-risk screening schedule (Papatheodoridi et al., 2021). Both indeterminate- (LSM: 8–12 kPa) and high-risk (LSM >12 kPa) patients should be referred to a hepatologist (Papatheodoridi et al., 2021). High-risk patients should begin treatment, while the indeterminate group may receive additional testing or monitor the condition with specialist supervision (Kanwal et al., 2021).

### Application of the NAFLD CCP

**Figure 2.**
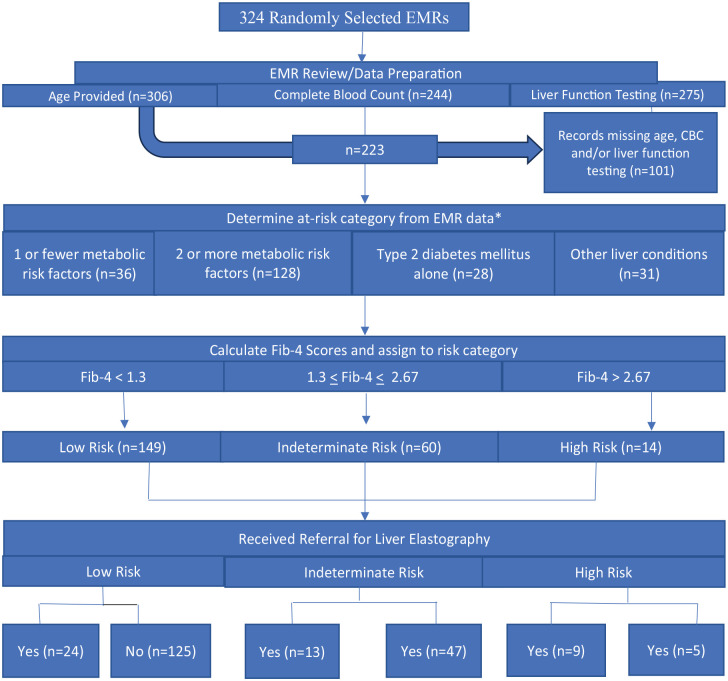
Retrospective Analysis Using AGA NAFLD CCP.

For this study, Steps 1 and 2 of the CCP screening process were carried out simultaneously to accommodate the retroactive application of the CCP (see Figure 2). Records missing essential laboratory values for this step (*n* = 83) represented the proportion of at-risk patients not appropriately screened for NAFLD at this stage. The remaining patients were divided into four categories based on risk type: one metabolic risk factor; two or more metabolic risk factors; type 2 diabetes with no comorbid metabolic factors; and other liver diseases (Kanwal et al., 2021). The CCP provided cut points to define metabolic risk factors. These include elevated serum triglycerides (≥150 m/dL), reduced serum high-density lipoprotein cholesterol (<40 mg/dL in men, <50 mg/dL in women), hypertension (systolic blood pressure ≥ 130 mmHg or diastolic blood pressure ≥ 85 mmHg) and elevated fasting glucose (plasma glucose > 100 mg/dL including prediabetes and diabetes) (Alberti et al., 2006; Kanwal et al., 2021). Assessment of the final metabolic risk factor, waist circumference, was impossible, as the metric was absent from the EMR. Although they do not meet CCP criteria to be considered at risk, patients with one metabolic risk factor were also included in this assessment as a comparison group. Finally, the “other liver disease” group comprised any patient with EMR documentation of nonviral liver abnormalities consistent with NAFLD/NASH (fatty liver, NASH, cirrhosis, abnormal AST/ALT, heavy alcohol use), regardless of metabolic comorbidities. During Step 2 of the pathway, patients with viral infections, clinical signs of advanced liver disease, and a history of heavy alcohol use would typically be excluded (Kanwal et al., 2021). Given the heterogeneous nature of this group’s “other liver conditions” and the retrospective nature of the study, which prevents us from knowing when in the process a diagnosis like NASH or cirrhosis would have been offered, this group was included across all steps of the CCP for comparison.

In staging liver disease risk in Step 3, first-visit laboratory values were used for the FIB-4 scores for each patient with complete data. Values that were out of the normal range were compared against follow-up laboratory values and replaced if there were significant differences. Platelets, AST, ALT levels, and age were used to calculate the FIB-4 score using the formula: age (years) × AST [U/L]/ (platelets [10^9^/L] × (ALT [U/L])^1/2^) (Sumida et al., 2014; Vallet-Pichard., 2007). Patients were then categorized as low (FIB-4 < 1.3), indeterminate (FIB-4 of 1.3–2.67), and high risk (FIB-4 > 2.67) based on their FIB-4 score. The FIB-4 score has a 94.7% negative predictive value and 74.3% sensitivity to exclude severe fibrosis with a FIB-4 score <1.45 and a positive predictive value of 82.1% and 98.2% specificity for FIB-4 score >3.25 when compared with liver biopsies (Vallet-Pichard et al., 2007; Lee et al., 2021; Balakrishnan et al., 2021). The next step in the CCP requires patients at indeterminate risk (FIB-4 = 1.3–2.67) to undergo liver elastography to assess liver stiffness.

Liver elastography is available at TTUHSC El Paso’s affiliate medical center, University Medical Center-El Paso (UMC). However, this contemporary technique only rose to popularity within the last few years and was unavailable to our study cohort. Standard radiological practices for fibrosis detection during the data collection period included upper right quadrant abdominal ultrasound, computed tomography (CT) scan, and MRI. In Step 4, the research team reviewed the EMRs to determine whether physicians referred patients for follow-up screening. Unfortunately, the EMRs did not include liver stiffness measurements, preventing the final stratification of the indeterminate patients among low-, indeterminate-, and high-risk groups per the CCP. In addition, though referral to specialists is an essential element of the CCP, it is not explored as an outcome in this introductory study as the records did not consistently record referrals or offer insight into the resultant follow-up care.

## Results

Table 1 displays EMR patient record demographic characteristics. The age distribution followed a bell-shaped curve, with the majority being 60–69 years old (27.3%). Female patients outnumbered males (59.2% vs. 40.8%), and 81.6% identified as Hispanic.

**Table 1.** Final Sample Demographic Characteristics (n = 223).

	*N*	%
Age		
Under 40	16	7.2
40–49	23	10.3
50–59	47	21.1
60–69	61	27.4
70–79	50	22.3
80+	26	11.7
Gender		
Male	91	40.8
Female	132	59.2
Ethnicity		
Hispanic/Latino	182	82.0
Not Hispanic/Latino	41	18.0

The at-risk categories employed in this study combined criteria from Steps 1 and 2 of the CCP. Based on the CCP criteria, 83.8% of patients in this sample were at risk for NAFLD. Over half of patients had two or more metabolic risk factors (57.4%), followed by type 2 diabetes (12.6%), then other liver conditions (13.9%). Only 16.4% did not meet the criteria, with only one metabolic risk factor. The average patient’s BMI was 32, and four patients were identified as having a history of heavy alcohol use. Table 2 details patient distribution across the at-risk categories.

**Table 2.** Patient Distribution by NAFLD Risks Factor and FIB-4 Risk Level (n=223).

Risk factor type	Total *n* (%)	Low risk *n* (%)	Intermediate risk *n* (%)	High risk *n* (%)
All NAFLD risk factors	223 (100.0)	149 (66.8)	60(26.9)	14 (6.3)
One or less metabolic risk factors	36 (16.1)	23 (63.9)	12 (33.3)	1 (2.8)
Two or more metabolic risk factors	128 (57.4)	84 (65.6)	32 (25.0)	12 (9.4)
Type 2 diabetes (only)	28 (12.6)	19 (67.9)	8 (28.6)	1 (3.6)
Pre-existing liver condition	31 (13.9)	23 (74.2)	8 (25.8)	0 (0.0)

Table 2 also presents overall FIB-4 risk-level results stratified by risk category. Based on the pathway’s FIB-4 cutoffs, 149 (66.8%) patients were considered low risk, 60 (26.9%) were indeterminate risk, and 14 (6.2%) were high risk. Distribution based on risk type indicated that patients with one metabolic risk factor had the highest percentage (33.3%) in the intermediate-risk FIB-4 category and the lowest in the low-risk FIB-4 category (63.9%). Patients with two or more metabolic risk factors had the highest percentage in the high-risk FIB-4 category (9.4%) and the lowest in the intermediate-risk category (25.0%). Patients with diabetes only and other liver diseases represented the highest group percentages in the low-risk FIB-4 category (67.0% and 74.2%, respectively).

The final step of the CCP is liver imaging for patients who are at intermediate risk. However, only 21.7% of patients with an indeterminate risk of NAFLD had records of receiving any follow-up imaging, most commonly upper right quadrant ultrasound (see Table 3). In addition, 35.7% of high-risk patients did not receive a referral for follow-up screening, while 16.1% of low-risk patients received an unnecessary one.

**Table 3.** Patient Distribution by NAFLD Risk Factor (CCP Step 1) (n = 223).

Liver imaging status	Low risk *n* (%)	Intermediate risk *n* (%)	High risk *n* (%)
Receive ultrasound or MRI	24 (16.1)	13 (21.7)	9 (64.3)
Did not receive ultrasound or MRI	125 (83.9)	47 (78.3)	5 (35.7)

## Discussion

The AGA designed the NAFLD CCP to increase NAFLD screening and improve timely treatment (Kanwal et al., 2021). Proactive screening in the primary care setting is essential for early detection of NAFLD and mitigation of disease progression to NASH or cirrhosis and its associated complications. An alarming 83.3% of patients in this study met the criteria for Step 1 of the CCP, meaning they were suspected to have NAFLD. The experimental NAFLD prevalence significantly exceeds the proportion observed in the U.S. population, which a 2018 meta-analysis of 33 studies found to range between 6.6% and 46.0%, with a pooled prevalence of 15.1% (Rich et al., 2018). It also significantly exceeded the prevalence rate of 22.4% observed in the U.S-based Hispanic population (Rich et al., 2018). In this study, Mexican American men with class II obesity had a higher prevalence of NAFLD (59.5%) compared to Mexican American women with class II obesity (52.2%) (Lazo et al., 2013). A more recent study of NHANES (2011–2018) using the U.S. Fatty Liver Index (USFLI) found that 64.6% of total Hispanic/Latinos had NAFLD, compared to non-Hispanic Whites (26.6%), non-Hispanic Black (24.3%), and non-Hispanic Asians (14.4%) (Wang et al., 2024). The high rate of suspected NAFLD in the study population was attributed to the enriched sample, which required the presence of at least one NAFLD risk factor. However, it is unlikely that enrichment alone can account for the alarmingly high rates of suspected NAFLD, indicating that unique population and environmental influences in this U.S–Mexico border setting contribute to an elevated risk.

Just over a third of our sample (33.2%) were at indeterminate (26.9%) or high risk (6.3%) for advanced fibrosis based on FIB-4 cutoffs 1.30 and 2.67, respectively, detailed in Step 3 of the CCP. The number of patients at indeterminate or high risk for NAFLD with advanced fibrosis was slightly above the rates of 18.6% and 1.4% observed in the National Health and Nutrition Examination Survey, which is representative of the U.S. population (Golabi et al., 2022). In addition, in an 11-study meta-analysis, the rate of advanced fibrosis in the Hispanic population was 19.4% (Rich et al., 2018). In addition to elevated prevalence, our study population also experiences more severe cases of NAFLD, placing an additional burden on patients and local medical systems. Findings support the consensus that U.S. Hispanics, particularly those of Mexican descent, experience high rates of NAFLD and emphasize the importance of screening in high-risk settings like U.S.–Mexico border towns (Fleischman et al., 2014; Garcia et al., 2022; Kallwitz et al., 2019).

Step 3 of the CCP pathway recommends that indeterminate-risk patients receive referrals for liver elastography to assess liver stiffness. Unfortunately, in the case of our study, liver elastography was not yet a widely available service. Instead, this study utilized other radiological techniques (ultrasound, CT, and MRI) as placeholders to conceptualize provider behavior. Most indeterminate-risk patients (78.3%) in our cohort did not receive a referral for follow-up imaging, especially those at risk due to two or more metabolic risk factors. About a third of the high-risk group and a few low-risk patients also received a liver imaging referral. In both cases, this represents an inefficient utilization of resources as the low-risk patients were referred to an unnecessary procedure, while high-risk patients required referral to specialist care. In addition to issues with referrals, there were evident shortcomings in laboratory testing that must be addressed. Given the high prevalence and elevated severity of NAFLD observed in our study population, there is a clear incentive to educate PCPs and nurses on the pathway, which will address the diagnostic shortcomings highlighted in this study. Implementation of the CCP in the primary care setting has the potential to detect NAFLD cases that may otherwise be overlooked. The standardized process ensures effective, resource-conscious screening and appropriate follow-up care while alleviating nurse and PCP’s decision-making burden.

### The Role of Nursing

The task force that created the CCP was divided into three teams: screening, diagnosis, and management (Kanwal et al., 2021), but it was not specified if a nurse was within the group. The primary objective of screening high-risk populations was to initiate early interventions and stop the progression of liver-related and all-cause mortality as well as cirrhosis (Kanwal et al., 2021). Oftentimes, nurses are in the greatest position to encourage an early investigation (Mortimore, 2022). Clinicians from a variety of specialties, such as primary care, gastroenterology, hepatology, obesity management, and endocrinology, are needed to co-manage the hepatic manifestations of the disease as well as the comorbid metabolic traits and cardiovascular risk to provide the best care possible for the expanding number of patients with NAFLD and NASH. To change unfavorable cardiometabolic risk factors, lifestyle interventions should be the focus of care for these patients (Kanwal et al., 2021). According to new research, involving nurses in the clinical management and care of patients with NAFLD improves patient outcomes and adherence to lifestyle modifications (Clayton et al., 2021). Multidisciplinary teams that include nurses also report reduced risk of cardiovascular disease, diabetes, metabolic health issues, liver enzyme levels, and liver stiffness (Clayton et al., 2021).

### Limitations

There are several limitations to this study. First, the data provided by the TTUHSCEP clinic was used as a proxy for the El Paso, Texas population and may misrepresent the region’s actual NAFLD burden. The patient population skews older, a population at increased risk of developing NAFLD, potentially contributing to an overestimation of NAFLD prevalence. Conversely, the missing EMR values may have underestimated NAFLD prevalence and affected accurate risk stratification. Waist circumference, a proxy for obesity, was unavailable, meaning patients with obesity and one other risk factor were likely excluded from the 2+ metabolic risk factor group. Metrics to calculate FIB-4 were also missing for 101 patients, leading to the omission of patients from the final analysis. Additional research is needed to determine the true prevalence of NAFLD among the El Paso population.

## Conclusion

The CCP is a feasible tool for NAFLD screening in primary care settings on the U.S.–Mexico border. This study’s findings indicate that patients at risk of NAFLD and its comorbidities could benefit significantly from CCP implementation in the primary care setting. Our results suggest that El Paso providers are familiar with NAFLD and make an effort to respond to the condition. However, primary care provider behaviors reflect inconsistent decision-making that results in inappropriate utilization of resources, culminating in suboptimal detection and treatment of NAFLD. Retrospective application of the CCP demonstrates the tool’s potential as a corrective guide capable of improving NAFLD detection and referral rates to appropriate screening and care, especially among indeterminate-risk patients. Given the pathway’s potential to reduce the detrimental impact of liver disease in border communities, there is a clear need for health provider education regarding the CCP.

Using this preliminary study as a guide, the research team plans to develop PCP and nurse education to familiarize them with CCP pathway guidelines and subsequently improve NAFLD risk detection and screening practices in El Paso. The effectiveness of this programming will be assessed with future prospective studies in which more rigorous hypothesis testing can be employed, and elements like referrals to testing and specialist care and their outcomes can be tracked reliably. A key component of the program would be to improve consonance between the health care beliefs of border residents and local health care systems. Cultures tend to have their own beliefs about health disease, treatment, and health care providers, and the U.S.–Mexico border is no different. Some obstacles that will have to be considered are the adherence to Mexican American folk customs and beliefs, how patients respond to specific therapies, and most importantly the use of Spanish. It is no surprise that people in this region could feel more comfortable speaking to a nurse who is also of Mexican descent and is well-versed in Spanish or is bilingual. For those who do not speak English, physicians and nurses will need to use verbal and nonverbal communication, which can be viewed as a sign of respect, or they can ensure to have translators on hand who are well-versed in the Mexican American form of Spanish. Further studies are needed to determine the prevalence and severity of NAFLD, by CCP standards, across diverse populations.
